# A Dual-Thread Lag–Locking Screw Enhances Single Lateral Plate Fixation in Bicondylar Tibial Plateau Fractures: A Biomechanical Study

**DOI:** 10.3390/bioengineering12101023

**Published:** 2025-09-25

**Authors:** Ya-Han Chan, Hsuan-Wen Wang, Wei-Che Tsai, Chun-Li Lin

**Affiliations:** 1 Department of Biomedical Engineering, National Yang Ming Chaio Tung University, Hsinchu 112304, Taiwan; helen19977@hotmail.com (Y.-H.C.); wayne.be11@nycu.edu.tw (W.-C.T.); 2 Medical Device Innovation & Translation Center, National Yang Ming Chiao Tung University, Hsinchu 112304, Taiwan

**Keywords:** schatzker, lag screw, locking plate, biomechanics, fatigue

## Abstract

Schatzker type V bicondylar tibial plateau fractures present a major challenge due to the difficulty of achieving stable fixation with minimally invasive strategies. This study introduces a dual-thread lag and locking plate (DLLP) design that integrates lag screw compression with unilateral locking plate fixation. A custom-built compression evaluation platform and standardized 3D-printed fracture models were employed to assess biomechanical performance. DLLP produced measurable interfragmentary compression during screw insertion, with a mean displacement of 1.22 ± 0.11 mm compared with 0.02 ± 0.04 mm for conventional single lateral locking plates (SLLPs) (*p* < 0.05). In static testing, DLLP demonstrated a significantly greater maximum failure force (7801.51 ± 358.95 N) than SLLP (6224.84 ± 411.20 N, *p* < 0.05) and improved resistance to lateral displacement at 2 mm (3394.85 ± 392.81 N vs. 2766.36 ± 64.51 N, *p* = 0.03). Under dynamic fatigue loading simulating one year of functional use, all DLLP constructs survived 1 million cycles with <2 mm displacement, while all SLLP constructs failed prematurely (mean fatigue life: 408,679 ± 128,286 cycles). These findings highlight the critical role of lag screw compression in maintaining fracture stability and demonstrate that DLLP provides superior biomechanical performance compared with SLLP, supporting its potential as a less invasive alternative to dual plating in the treatment of complex tibial plateau fractures.

## 1. Introduction

Tibial plateau fractures are critical injuries that compromise the stability and function of the knee joint, particularly in Schatzker type V bicondylar fractures, where both the medial and lateral columns are damaged and the joint surface is comminuted [1,2,3,4,5]. These fractures require highly stable internal fixation to restore biomechanical function and promote bone healing. Traditionally, dual locking plates (DLPs) have been considered the preferred surgical treatment for such fractures, effectively controlling medial fragment displacement and providing multidirectional rigidity [6,7,8,9,10,11,12]. However, the extensive soft tissue detachment, limited positioning of bone screws, prolonged surgical time, and increased risk of infection associated with the bilateral approach have led to growing interest in the minimally invasive and biocompatible single lateral locking plate (SLLP) strategy [6,7,8,9].

The SLLP offers a less invasive alternative, and several studies have confirmed its favorable clinical outcomes in selected cases [6,7]. Nevertheless, in the presence of a posteromedial fragment, SLLP alone may fail to provide sufficient compression and medial column stability, increasing the risk of secondary displacement, delayed collapse, or nonunion [6,7,8,9,10,11,12]. This limitation arises because currently available commercial locking plates are paired only with traditional locking screws, which offer limited mechanical stability and reduction effectiveness for double-column fracture configurations with displacement risk.

To address this challenge, integrating a lag screw into an SLLP instead of a traditional screw can provide additional compressive force and stable support without increasing the medial approach, thereby enhancing fixation of the medial bone fragment. This innovative approach not only improves the biomechanical stability of the fracture site but also minimizes the risk of complications associated with soft tissue damage. A lag screw achieves interfragmentary compression through its design—characterized by a threaded distal segment, a smooth sliding shaft, and a variable pitch—that generates axial compression during insertion, promoting bone fragment apposition and construct rigidity [13,14,15,16,17]. When applied to Schatzker type V bicondylar fractures, a lag screw with differential thread pitches on the medial and lateral ends, combined with a central sliding interface, can potentially integrate both compression and locking functions. This dual-thread lag and locking plating (DLLP) design holds considerable promise; however, its biomechanical efficacy within the SLLP construct has not yet been systematically validated.

This study introduces a DLLP that integrates lag screw compression with unilateral locking plate fixation, enabling active interfragmentary compression while preserving the angular stability of a locking construct. A customized compression-assessment platform was further developed to directly quantify interfragmentary compression during screw insertion, addressing a methodological gap in prior research that focused only on overall construct stability. In addition, standardized 3D-printed fracture models reconstructed from clinical imaging ensured reproducibility and consistency in static and fatigue testing [18,19,20,21,22]. Together, these innovations provide systematic biomechanical evidence that screw-induced compression is a critical mechanism for enhancing stability in minimally invasive single-plate constructs.

This study hypothesized that the DLLP could generate greater interfragmentary compression and superior biomechanical stability compared with conventional SLLP, particularly in managing the posteromedial fragment of bicondylar tibial plateau fractures. The objective of this study was therefore to evaluate and compare the static and dynamic biomechanical performance of DLLP and SLLP fixation using standardized 3D-printed fracture models.

## 2. Materials and Methods

### 2.1. Compression Capability Evaluation of Screw Insertion

This study aimed to evaluate whether DLLPs and traditional SLLPs can effectively achieve compression during bone insertion. The testing group included DLLPs with a diameter of 4.0 mm, a total length of 75 mm, and proximal and distal thread pitches of 0.7 mm and 1.05 mm, respectively. These screws were paired with shafts of 13.5 mm and 17 mm in length, respectively, and contained a smooth, toothless central section. The control group included SLLPs with the same diameter, a total length of 70 mm, and a fully threaded design with a 1.0 mm pitch. Both screws were components of the APLS Asia Metal Locking Screw and Plate System (A Plus Biotechnology Co., Ltd., New Taipei City, Taiwan) and were made of Ti6Al4V titanium alloy with no special surface treatment.

**Figure 1:**
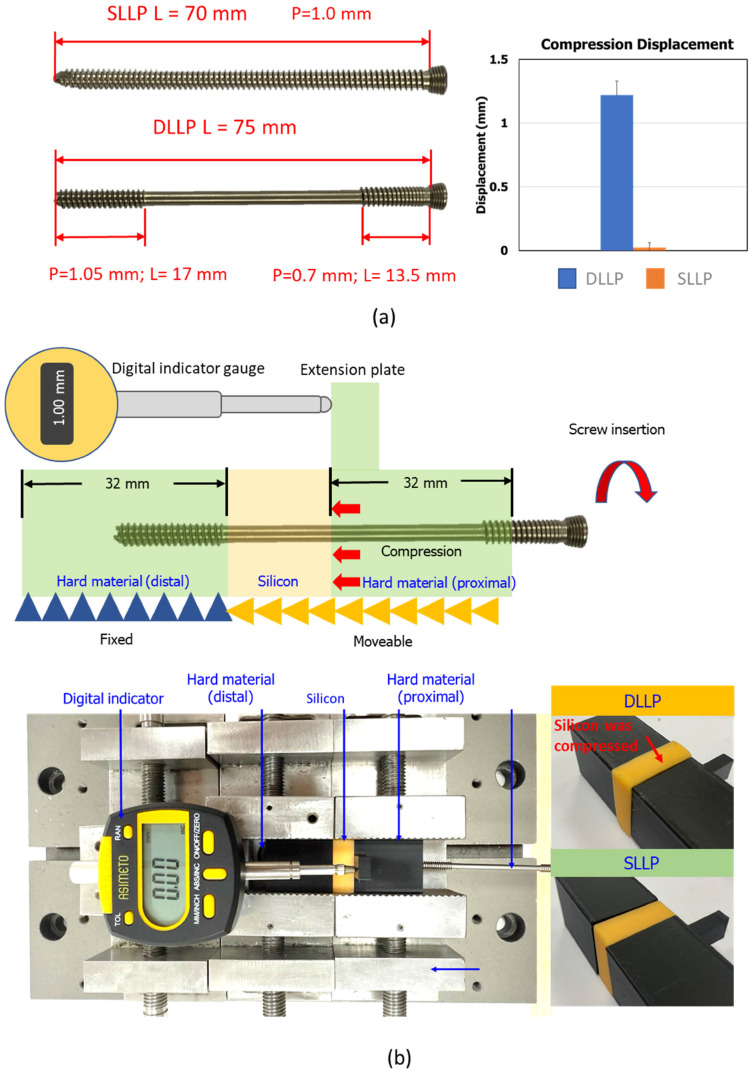
(**a**) Left: screw design; Right: DLLP insertion produced significantly greater displacement of the proximal block compared with SLLP, indicating effective compression generation. (**b**) Testing setup of the compression evaluation platform. A three-layer structure consisting of hard material blocks (distal fixed, proximal moveable) and a deformable silicone layer was used, with compression displacement measured by a digital indicator. Photographs show evident compression of the silicone layer with DLLP insertion, whereas SLLP produced negligible deformation.

To simulate the compression effects of screws used in tibial plateau fractures, a three-layer structure with pre-drilled holes was used. The proximal and distal ends consisted of hard material blocks measuring 32 mm × 25 mm × 25 mm, produced by PLA 3D printing (Figure 1). A 20 mm high extension plate was placed above the proximal block to facilitate subsequent measurement using an indicator probe (Figure 1). The center layer consisted of a deformable silicone material, Elite^®^ Double Fast 22 (Zhermack S.p.A., Badia Polesine, Italy), with the following properties: hardness (Shore A) = 22, load at break = 2.5 N/mm^2^, tear resistance = 5 N/mm^2^, and elongation at break = 450%. Using a special mold, a 1:1 mixture of the Double Fast 22 base and catalyst was prepared to produce a 25 mm × 25 mm × 10 mm rectangular block with a Ø3 mm cylindrical hole in the center.

After the materials were prepared, the three components were assembled along the same axis on the experimental platform. The distal hard block was fixed in a vise, while the silicone layer and the proximal material block were placed in the vise but not clamped on the sides. This setup prevented rotation due to torque during screw insertion and allowed the blocks to slide toward the distal block (Figure 1). Once the setup was complete, the probe tip of the digital indicator gauge (ASIMETO Series 405-95-0, resolution 0.01 mm, Mitutoyo Corporation, Kanagawa, Japan) was adjusted to contact the extension plate of the proximal block and then zeroed. The screw was then inserted into the three-layer structure (Figure 1b) using an electric drill until the front end of the locking head engaged. Five insertion tests were performed for each group (DLLP and SLLP), and compression was assessed by measuring the displacement of the proximal hard material block on the indicator gauge.

### 2.2. Fabrication of Biomechanical Test Specimens for Tibial Plateau Fracture Reduction

**Figure 2:**
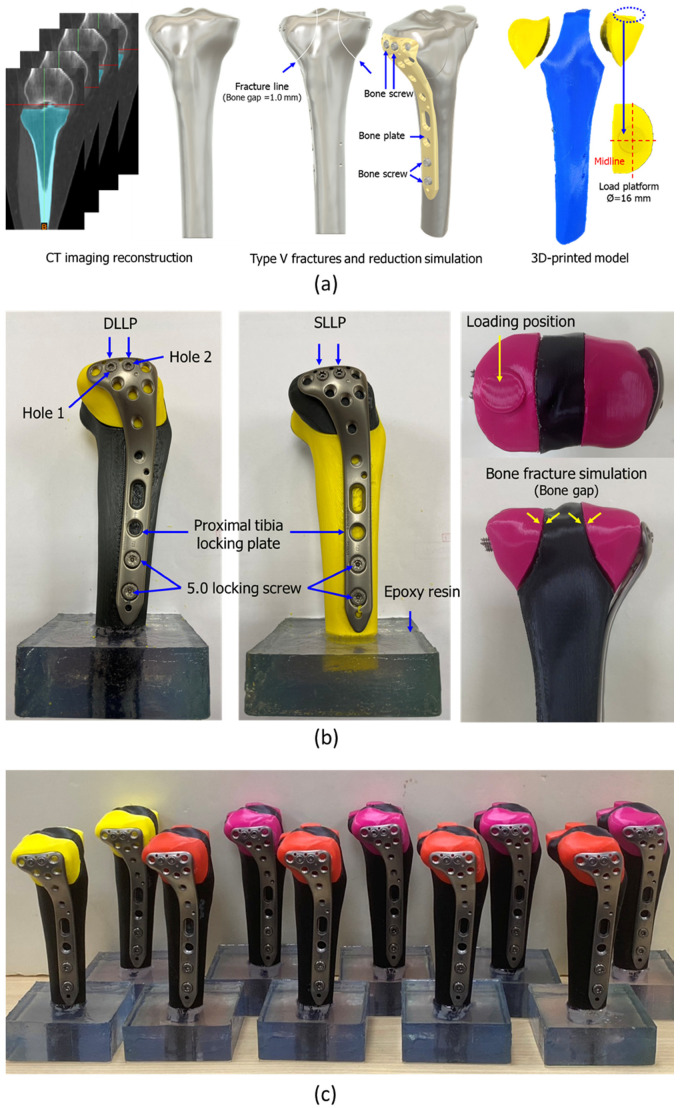
(**a**) CT imaging reconstruction, Schatzker type V fracture line design (1.0 mm gap), reduction simulation with bone plate and screws, and conversion into a 3D-printed fracture model with a 16 mm medial load platform. (**b**) Testing setup of fracture fixation. DLLP and SLLP screws were implanted in holes 1 and 2 of the proximal tibia locking plate, supplemented with 5.0 mm locking screws in the tibial shaft. Models were embedded in epoxy resin, and fracture gaps were maintained for loading simulation. (**c**) Completed 3D-printed tibial plateau fracture specimens fixed with locking plates and screws, prepared for static and dynamic biomechanical testing.

This test aimed to evaluate the fixation effects of two different bone screws after reduction of a tibial plateau fracture. Tibial CT images from a 66-year-old female patient obtained from previous research were used as the basis for specimen fabrication. (Taipei Medical University Joint Institutional Review Board (TMU-JIRB) Approval No.: N202006034) [23]. The DICOM image file was reconstructed into a three-dimensional model (Figure 2a) using reverse engineering software (Mimics 22.0, Materialise NV, Leuven, Belgium) and then imported into CAD software (Version 2021 R2, SpaceClaim, SpaceClaim Corporation, Concord, MA, USA) to reconstruct the solid model. The medial and lateral plateau fracture segments, with 1 mm fracture gaps, were generated according to Schatzker type V classification (Figure 2a). At the same time, the positions of the bone screws and plate were simulated in the software to generate the pre-drilling paths of the screws, ensuring positional consistency in subsequent experimental models. In addition, a 16 mm diameter cylindrical platform (Figure 2b) was designed on the medial side of the tibial plateau at the same location where force would be applied during the mechanical test (Figure 2a).

The reconstructed fracture model was 3D-printed using PLA (Flashforge Adventurer 5M, Zhejiang Flashforge 3D Technology Co., Ltd., Jinhua, China) with a layer thickness of 0.2 mm, an infill density of 100%, and a nozzle temperature of 210 °C. PLA was selected because of its consistent mechanical properties (elastic modulus: 1000–1100 MPa, ISO 527) and high reproducibility in fracture model production [24].

A lateral Ti6Al4V bone plate (length: 130 mm, model: 0702-0101-04, A Plus Biotechnology Co. Ltd., New Taipei City, Taiwan) with bone screws was selected to repair the fracture. For fixation at the tibial plateau, two types of screws were used: a 4.0 mm diameter SLLP (70 mm) and a DLLP (75 mm), implanted in holes 1 and 2, respectively, replacing the original four screws. Two additional locking screws, each 5.0 mm in diameter, were implanted into the tibial shaft (Figure 2b). After fracture reduction, the fracture gap was controlled between 0.5 and 1.2 mm (Figure 2b). The bottom of each specimen was embedded in epoxy resin (Figure 2b) for subsequent mechanical testing, with an embedding height of 30 mm from the bottom of the tibial shaft. The printed tibia models with fracture fixation by plate and screws are shown in Figure 2c.

### 2.3. Static and Dynamic Biomechanical Tests of Tibial Plateau Fracture Reduction

The specimens were divided into static and dynamic groups for SLLP and DLLP fixations (*n* = 3), with identical installation methods used for both tests. The sample size (*n* = 3 per group for static and fatigue tests) was determined based on previous biomechanical evaluations of tibial plateau fixation constructs, which reported adequate statistical power using 3–5 specimens per condition [6,25]. This approach ensured reproducibility while balancing resource feasibility.

**Figure 3:**
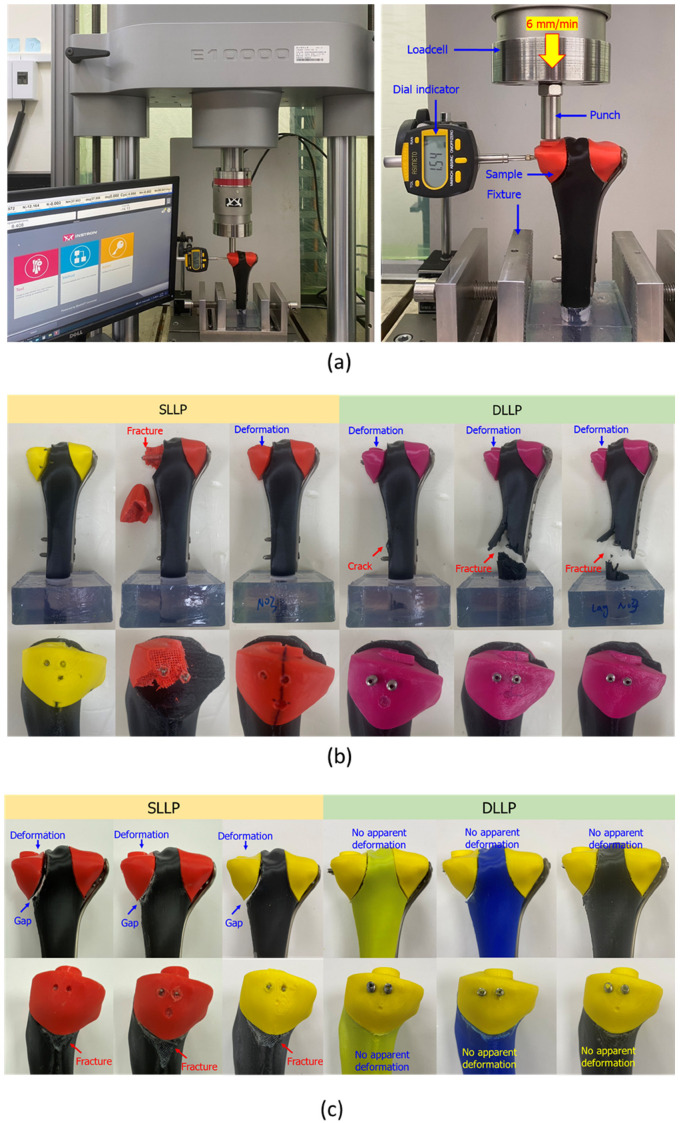
(**a**) Testing setup for static/dynamic compression testing using a testing machine. Axial load was applied at a displacement rate of 6 mm/min, with lateral displacement simultaneously measured by a dial indicator. (**b**) Failure modes after static testing. SLLP specimens exhibited medial bone block subsidence, deformation, and fracture, whereas DLLP specimens showed medial segment deformation and fracture with higher load transfer to the distal shaft. (**c**) Failure modes after dynamic fatigue testing. SLLP specimens demonstrated progressive deformation, gap widening, and fracture, while DLLP specimens showed no apparent deformation or fracture after 1,000,000 loading cycles.

A digital indicator gauge was mounted on the medial side of the proximal tibial plateau to measure lateral displacement during testing. The applied machine load and indicator readings were simultaneously recorded using a video recorder, as shown in Figure 3a. Static testing was performed under displacement-controlled loading at a rate of 6 mm/min, and the test was terminated when specimen failure occurred, defined as a 20% reduction in maximum force.

The dynamic loading was determined based on the tibial force distribution during human gait reported by Wehner et al. [26], in which the maximum tibial plateau load reached approximately 3.3 times body weight. Assuming a body weight of 80 kg, the forces were distributed as 60% on the medial tibial plateau and 40% on the lateral plateau. Accordingly, the load applied to the medial tibial plateau was calculated as 80 kg × 9.8 N/kg × 60% × 3.3 = 1552.32 N.

Dynamic fatigue testing was conducted using an electro-mechanical testing machine (Instron ElectroPuls E10000, Instron Corp., Norwood, MA, USA). Each specimen was fixed vertically with the distal tibia embedded in epoxy resin to a depth of 30 mm, while axial load was applied through a 16 mm cylindrical platform positioned on the medial tibial plateau to simulate the physiological loading location. A sinusoidal waveform cyclic load ranging from 155.23 N to 1552.32 N (R ratio = 10) was applied at a frequency of 5 Hz. This loading regime corresponded to approximately one year of functional activity, equivalent to one million cycles, as suggested by Griffin et al. [27]. Throughout testing, axial displacement was continuously recorded by the machine, while lateral displacement was measured at intervals using a digital indicator mounted at the medial side of the tibial plateau. Failure was defined, consistent with Diffo et al. [28], as a tibial plateau displacement greater than 2 mm.

## 3. Results

In the compression capability evaluation test, both the SLLP and DLLP constructs were compressed toward the distal end. The average displacement (±SD) was 0.02 ± 0.04 mm for SLLP and 1.22 ± 0.11 mm for DLLP. Statistical analysis using a t-test showed a significant difference between the two groups (*p* < 0.05) (Figure 1a). The detailed deformation of the silicone material after testing is shown in Figure 1b. Noticeable swelling of the outer contour was observed when the DLLP was fixed in position, whereas the SLLP exhibited no obvious deformation or bulging after fixation.

**Figure 4:**
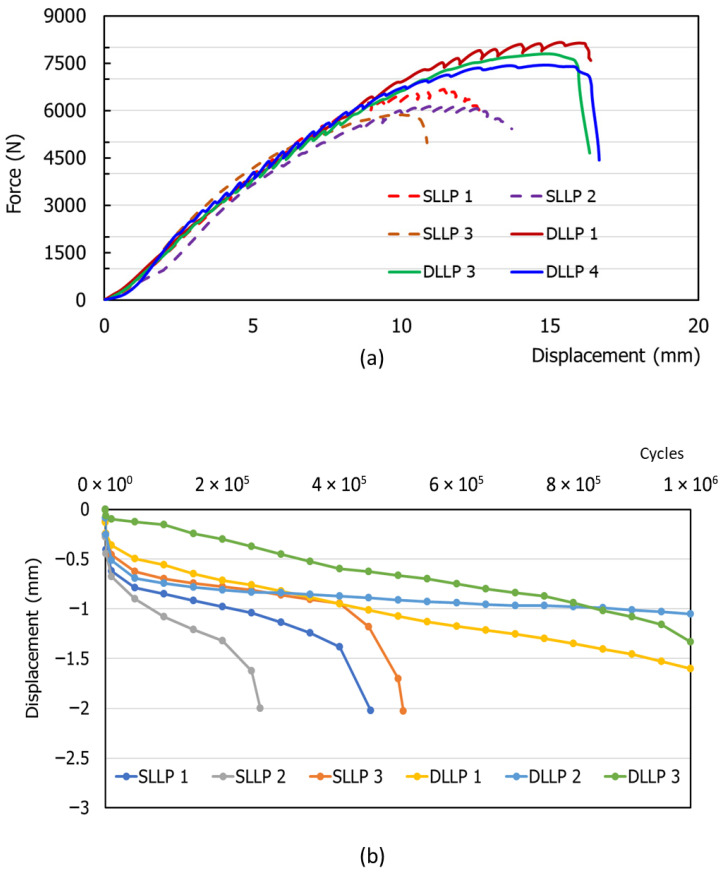
(**a**) Force–displacement curves of 3D-printed tibial plateau fracture models fixed with SLLP and DLLP constructs under static compression testing. DLLP specimens (solid lines) demonstrated higher maximum failure forces and greater displacement tolerance compared with SLLP specimens (dashed lines), indicating improved static fixation strength. (**b**) Axial displacement of tibial plateau fracture models fixed with SLLP and DLLP constructs during dynamic fatigue testing (up to 1,000,000 cycles). All DLLP specimens (yellow, blue, green lines) maintained displacement < 2 mm throughout the test, whereas all SLLP specimens (blue, gray, orange lines) failed prematurely before 1,000,000 cycles, indicating superior fatigue resistance of DLLP fixation.

Table 1 and Figure 4a present the results of the static compression tests on 3D-printed tibial plateau fracture models stabilized with SLLP and DLLP. The mean maximum failure force of DLLP was 7801.51 ± 358.95 N, significantly higher than that of SLLP (6224.84 ± 411.20 N, *p* < 0.05). At an axial displacement of 2 mm (measured directly by the testing machine), the mean force for DLLP was 1515.93 ± 78.62 N compared with 1329.17 ± 330.44 N for SLLP; however, this difference was not statistically significant (*p* = 0.20). At a lateral displacement of 2 mm (measured by the digital indicator), the mean force was 3394.85 ± 392.81 N for DLLP and 2766.36 ± 64.51 N for SLLP, with the difference reaching statistical significance (*p* = 0.03). Figure 3b illustrates the failure modes of the repaired tibial plateau fracture system after testing. In the SLLP group, failures were mainly observed in the medial fracture segment, with two specimens showing distal (downward) deformation and one exhibiting segment fracture. In contrast, all DLLP specimens demonstrated distal deformation of the medial fracture segment, and due to the higher destructive force sustained by the tibial plateau, subsequent damage extended to the distal bone shaft (fractures in two specimens and pronounced cracking in one).

During the dynamic test, axial displacement was continuously recorded in real time by the testing machine, allowing the relationship between load cycles and axial displacement to be obtained (Figure 4b). Data were extracted at the 1st, 1000th, 10,000th, and 50,000th cycles and then at every 50,000th cycle up to 1,000,000. After completion, lateral displacement was measured using a digital indicator. If failure occurred before 1,000,000 cycles, the corresponding cycle number as well as axial and lateral displacements were recorded.

The results showed that all three SLLP specimens failed before 1,000,000 cycles, with a mean fatigue life of 408,679 ± 128,286 cycles. At failure, the average axial and lateral displacements were 2.017 ± 0.013 mm and 1.377 ± 0.093 mm, respectively. In contrast, all three DLLP specimens endured beyond 1,000,000 cycles, with final axial and lateral displacements of 1.330 ± 0.274 mm and 0.973 ± 0.119 mm, both below the 2 mm threshold (Table 2).

Figure 3c illustrates the failure modes of the repaired tibial plateau fracture system after dynamic testing. SLLP specimens exhibited pronounced medial bone segment deformation with enlarged cracks and damage to the distal bone shaft (including material detachment). In contrast, DLLP specimens showed no obvious bone segment damage or displacement.

## 4. Discussion

This study proposed a novel design combining lag screw compression with unilateral locking plate fixation (DLLP) for Schatzker type V two-column tibial plateau fractures. By integrating a custom compression platform and 3D-printed fracture models, we systematically verified both the feasibility and biomechanical advantages of DLLP compared with conventional SLLP constructs.

The specially designed compression platform in this study provided a unique methodological advantage by directly demonstrating the interfragmentary compression generated during screw insertion, rather than only assessing overall construct stability as in conventional biomechanical tests. Using this platform, DLLP produced measurable displacement of the proximal block toward the distal end (1.22 ± 0.11 mm) and visible deformation of the silicone layer, whereas SLLP showed negligible compression (0.02 ± 0.04 mm, *p* < 0.05). This direct visualization and quantification not only verified the theoretical design concept of DLLP but also offered mechanistic validation that its lag component effectively transmits compressive force across the fracture gap, highlighting the practical benefit of achieving immediate interfragmentary compression without additional medial approaches. Beyond this study, the platform also provides a versatile tool for systematically evaluating the compression performance of different lag screw designs, thereby facilitating implant optimization and accelerating the translation of novel screw concepts into clinical applications.

In parallel, the standardized 3D-printed Schatzker type V fracture models ensured reproducibility of fracture geometry, screw positioning, and loading conditions, allowing consistent and reliable comparison of fixation strategies. Although combining lag screw compression with the fixation advantages of a locking plate is not a new concept in orthopedic surgery, systematic biomechanical validation has been limited by the difficulty of reliably replicating such complex fracture patterns in cadavers or sawbone models. Advances in CT image reconstruction and 3D-printing technologies now make it possible to create standardized and reproducible fracture models, thereby overcoming these challenges and improving both the reliability of comparisons and the feasibility of trend analysis [29,30,31]. While mechanical differences remain between PLA and real bone, these models still provide valuable experimental platforms for comparative evaluation of fixation methods.

The DLLP employs different thread pitches at the proximal and distal ends with a smooth central section, enabling the screw to generate a compression effect across the fracture site during insertion. The compression test confirmed that DLLP produced measurable displacement of the proximal block toward the distal end, verifying its effective compression mechanism. This innovative design not only enhances initial stability but also promotes favorable healing conditions at the fracture site. Importantly, delayed screw compression plays a crucial role in maintaining long-term stability by sustaining interfragmentary contact during cyclic loading. Unlike SLLP constructs, which lack active compression and therefore allow progressive micromotion and gap widening, DLLP provides a continuous compressive preload that reduces micro-gaps, improves load sharing between medial and lateral fragments, and delays secondary collapse. These findings are consistent with the report by Jiang et al. (2024), who demonstrated that cancellous lag screws increased interfragmentary compression force and improved structural stability in osteoporotic tibial plateau fractures under both cyclic loading and finite element analysis [25]. Collectively, this evidence highlights the critical role of lag screw compression—both immediate and delayed—in maintaining fracture-end stability and improving long-term biomechanical performance.

In this study, fracture segment displacement exceeding 2 mm during static and dynamic mechanical testing was defined as fixation failure. This criterion has been widely used in clinical and biomechanical research, as articular surface displacement greater than 2 mm can lead to joint incongruity, which in turn contributes to articular cartilage degeneration and post-traumatic arthritis [25]. Therefore, the 2 mm threshold is not only clinically meaningful but also provides a consistent and quantifiable benchmark for comparing fatigue tolerance across different fixation strategies.

The results of static biomechanical test showed that DLLP achieved a mean maximum failure force of 7801.51 ± 358.95 N, significantly higher than 6224.84 ± 411.20 N for SLLP (*p* < 0.05), and exhibited greater resistance to 2 mm lateral displacement (3394.85 ± 392.81 N vs. 2766.36 ± 64.51 N, *p* = 0.03). Failure mode analysis further revealed that SLLP constructs were prone to subsidence or fragmentation of the medial bone block, whereas DLLP, due to its greater load-bearing capacity, shifted the weakness to the distal bone shaft, where cracks or fractures occasionally occurred—consistent with prior reports that reinforcing medial support redistributes stress to other regions [25]. However, these macroscopic failure patterns, including subsidence, fragmentation, and distal shaft cracks, also indirectly reflect the presence of non-uniform deformation within the constructs. Although quantitative strain-field mapping was not performed in this study due to the use of PLA models, the observed fracture morphologies illustrate how stress concentration and uneven load transfer contributed to construct failure. Moreover, dynamic fatigue testing confirmed that all DLLP specimens survived 1,000,000 cycles with displacement maintained below 2 mm, while all SLLP specimens failed prematurely with a mean fatigue life of 408,679 ± 128,286 cycles. Collectively, these findings provide robust data support for the superior biomechanical performance and fatigue resistance of DLLP under long-term loading.

The effectiveness of DLLP depends largely on whether the screw is long enough to cross the fracture site and ensure that the proximal and distal threads are anchored on both sides of the fracture, thereby effectively generating compressive force and improving fixation strength. If the screw is not sufficiently long or is improperly positioned, the lag screw will not function as intended. This is consistent with the findings of Jiang et al. [25], who reported that if the lag screw is not anchored across the fracture site, its contribution to interfragmentary compression and overall stability is greatly reduced. Therefore, in clinical practice, careful preoperative planning and intraoperative image guidance are essential to ensure the correct screw length and positioning.

From a clinical perspective, the DLLP concept may offer a less invasive alternative to dual plating for Schatzker type V bicondylar tibial plateau fractures by integrating lag screw compression with lateral plate fixation, thereby enhancing medial column stability while reducing soft tissue dissection and surgical morbidity. However, several limitations should be acknowledged. This study was conducted using PLA-based 3D-printed models that cannot fully replicate the complex biomechanical properties of human bone, the sample size was limited, and direct comparisons with dual plating—the current clinical gold standard—were not performed. Future investigations should therefore incorporate cadaveric or large-animal models to verify whether DLLP can achieve comparable or superior biomechanical performance, explore its effectiveness under osteoporotic conditions, and ultimately confirm its clinical applicability through prospective trials.

## 5. Conclusions

This study demonstrated the biomechanical advantages of the dual-thread lag–locking plate (DLLP) construct over conventional single lateral locking plate (SLLP) fixation in Schatzker type V tibial plateau fractures:

1. Immediate compression: DLLP generated measurable interfragmentary compression during screw insertion (mean displacement: 1.22 ± 0.11 mm) compared with negligible compression for SLLP (0.02 ± 0.04 mm, *p* < 0.05).
2. Static strength: DLLP exhibited a significantly greater maximum failure force (7801.51 ± 358.95 N) than SLLP (6224.84 ± 411.20 N, *p* < 0.05) and higher resistance to lateral displacement at 2 mm (3394.85 ± 392.81 N vs. 2766.36 ± 64.51 N, *p* = 0.03).
3. Fatigue resistance: All DLLP constructs withstood 1,000,000 cycles, with displacement maintained below the clinical threshold, while all SLLP constructs failed prematurely, with a mean fatigue life of 408,679 ± 128,286 cycles.
4. Failure modes: SLLP tended to cause medial fragment subsidence or fragmentation, whereas DLLP, due to its greater stability, transferred failure to the distal tibial shaft, reflecting improved medial support.

Overall, DLLP effectively combines lag screw compression with locking plate fixation, providing superior initial fixation strength and long-term stability. It represents a promising, less invasive alternative to dual plating for complex bicondylar tibial plateau fractures.

## Figures and Tables

**Table 1:** Results of static biomechanical tests of tibial plateau fracture reduction.

Static Test	Max. Force (N)	Axial 2 mm Disp. Force (N)	Lateral 2 mm Disp. Force (N)
SLLP-No1	6674.59	1404.80	2828.10
SLLP-No2	6131.82	967.47	2699.40
SLLP-No3	5868.12	1615.24	2771.57
Average (Std.)	6224.84 (411.20)	1329.17 (330.44)	2766.36 (64.51)
DLLP-No1	8161.21	1549.31	3083.02
DLLP-No2	7800.00	1426.13	3265.50
DLLP-No3	7443.31	1572.36	3836.02
Average (Std.)	7801.51 (358.95)	1515.93 (78.62)	3394.85 (392.81)
*t*-test	*p* = 0.00 *	*p* = 0.20	*p* = 0.03 *

* Indicates significant differences (*p* < 0.05) between groups.

**Table 2:** Results of dynamic biomechanical tests of tibial plateau fracture reduction.

Dynamic Test	Total Cycle	Max. Axial Disp. (mm)	Max. Lateral Disp. (mm)
SLLP-No1	453,349	2.021	1.27
SLLP-No2	264,029	2.002	1.42
SLLP-No3	508,658	2.028	1.44
Average (Std.)	408,679 (128,286)	2.017 (0.013)	1.377 (0.093)
DLLP-No1	1,000,000	1.601	1.11
DLLP-No2	1,000,000	1.054	0.92
DLLP-No3	1,000,000	1.334	0.89
Average (Std.)	1,000,000	1.330 (0.274)	0.973 (0.119)

## Data Availability

The data presented in this study are available on request from the corresponding author.
